# Paraneoplastic Pemphigus Mimicking Pemphigus Vulgaris Associated With Castleman Disease

**DOI:** 10.7759/cureus.36114

**Published:** 2023-03-14

**Authors:** Mariana Grigore, Mariana Costache, Olga Simionescu

**Affiliations:** 1 Department of Dermatology I, Colentina Clinical Hospital, Carol Davila University of Medicine and Pharmacy, Bucharest, ROU; 2 Department of Pathology, Emergency University Hospital, Carol Davila University of Medicine and Pharmacy, Bucharest, ROU

**Keywords:** unicentric castleman disease, paraneoplastic pemphigus, castleman’s tumor, autoimmune bullous dermatoses, pemphigus vulgaris

## Abstract

Paraneoplastic pemphigus (PNP) is a rare bullous disease with a polymorphic presentation. Diagnosis can be difficult because it can mimic other bullous diseases, while the underlying neoplasm may be completely asymptomatic. We present the case of a 19-year-old female with a four-year history of exclusively oral bullous lesions, mimicking pemphigus vulgaris, before the diagnosis of a retroperitoneal Castleman disease. While PNP is a severe and sometimes deadly condition, our patient had a mild and long evolution on minimal treatment, with complete resolution after tumor excision. Practitioners should be aware of PNP in young patients presenting with bullous disease and should conduct prompt systemic investigations in refractory or long-evolving cases, even when PNP diagnostic criteria are not fully met.

## Introduction

Paraneoplastic pemphigus (PNP) is a rare and often deadly autoimmune bullous disease associated with an underlying malignancy. It is most frequently associated with lymphoproliferative disorders, both malignant and benign [1]. Castleman disease (CD) is a rare benign lymph node hyperplasia of unknown etiology and is associated with PNP in 18% of cases [2]. Patients between 45 and 60 years of age are usually affected, but those with PNP and CD are younger and have a better prognosis, especially in the case of treatable unicentric CD [3].

No formal diagnostic or treatment protocols exist for PNP [4]. Diagnosis can be difficult as clinical and histological presentation is extremely polymorphous. Intractable stomatitis is the most characteristic clinical sign, but flaccid bullae, erosions, tense bullae, vesicles, and even lichenoid papules or targetoid lesions may be present. Antibodies against plakin family antigens are key laboratory findings, but cases may present with antibodies to many other antigens, such as desmoglein (DSG) 1 and 3 or bullous pemphigoid antigen (180 kDa). There are even reported cases of PNP with only anti-DSG 3 autoantibodies present [5]. The most characteristic histological findings are acantholysis and interface dermatitis, but histology may mimic other blistering and non-blistering diseases. High-dose corticosteroids are usually the first line of treatment along with corticoid-sparing agents and tumor excision when possible. Here, we present a case of PNP clinically and histologically mimicking mucous pemphigus vulgaris in association with a retroperitoneal CD in a 19-year-old female, with a four-year-long evolution and excellent response to treatment.

## Case presentation

**Figure 1:**
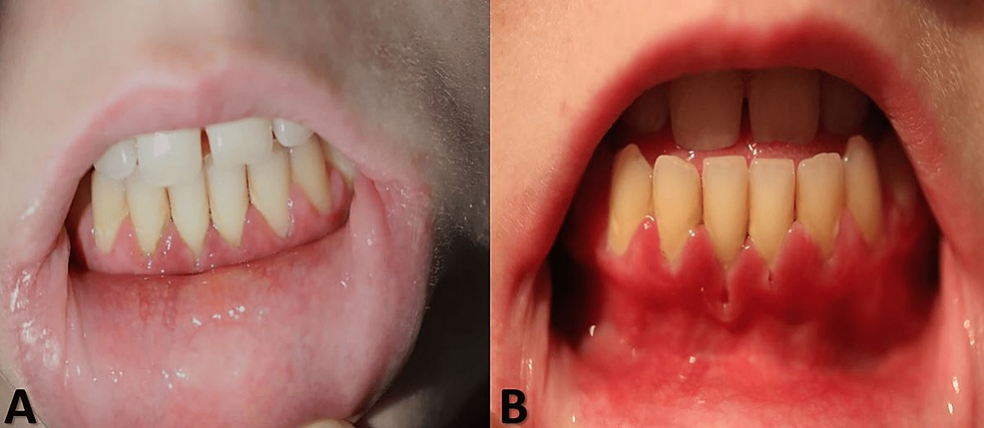
Clinical presentation. Erosive lesions on the inner lower lip (A) with gingival involvement and retraction (B).

**Figure 2:**
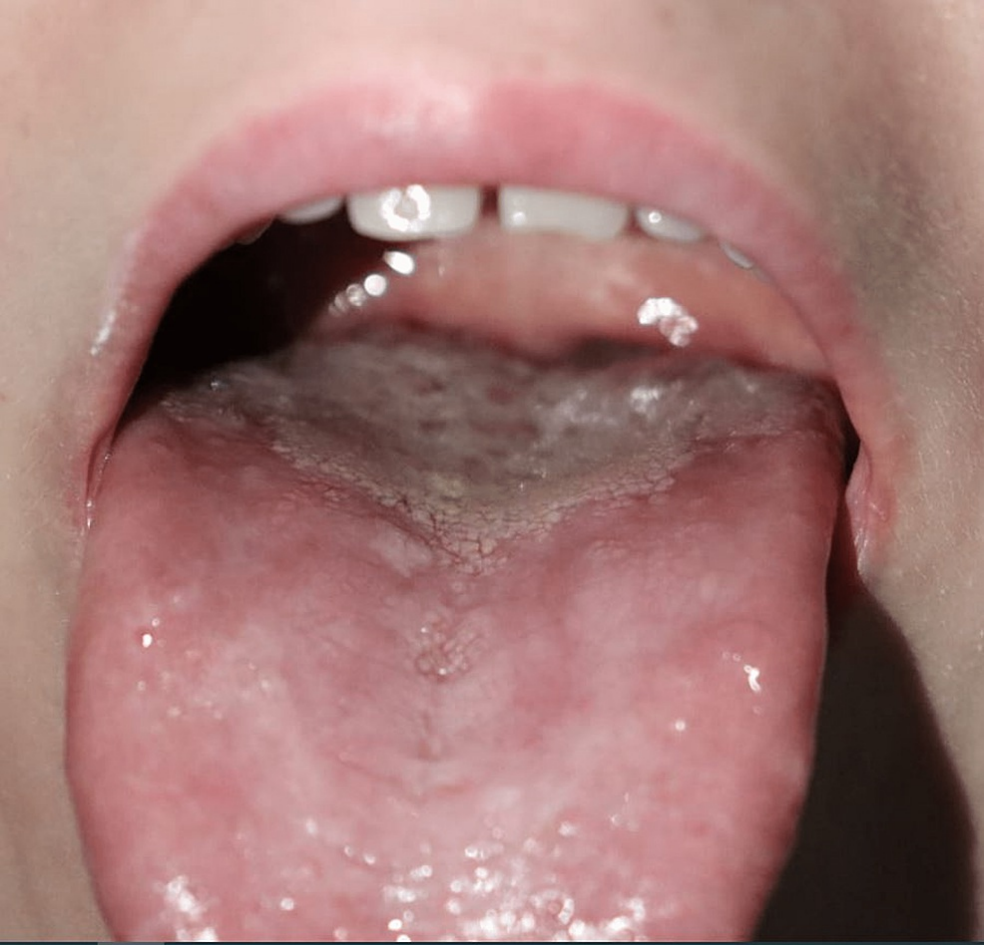
Clinical presentation. Glossitis with depapillation of the dorsal surface of the tongue.

A 19-year-old female presented to our clinic for a second opinion concerning painful erosive oral enanthema in August 2018. History revealed one and a half years of evolution before presentation, with previous investigations failing to establish a definitive diagnosis. She had an inconclusive biopsy, negative direct immunofluorescence (DIF), negative mycological examination, and a faint positive antinuclear antibody (ANA) test. She had been treated discontinuously with low-dose prednisone but was off any systemic treatment at the time of presentation. Upon clinical examination, the patient had painful oral erosive lesions (gingiva, inner lips, soft palate, pharynx), glossitis with depapillation of the tongue, and dysphagia (Figures 1, 2). No skin lesions or involvement of other mucous sites were noted. The patient had no other symptoms or complaints, and the general examination, personal history, and family history were all unremarkable.

**Figure 3:**
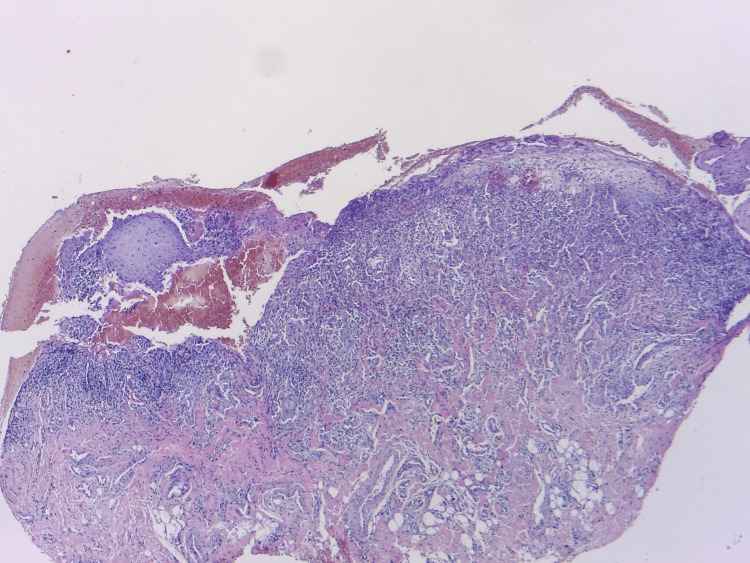
Histology examination (hematoxylin and eosin stain, 40×). Epidermic bullae with suprabasal acantholysis.

**Figure 4:**
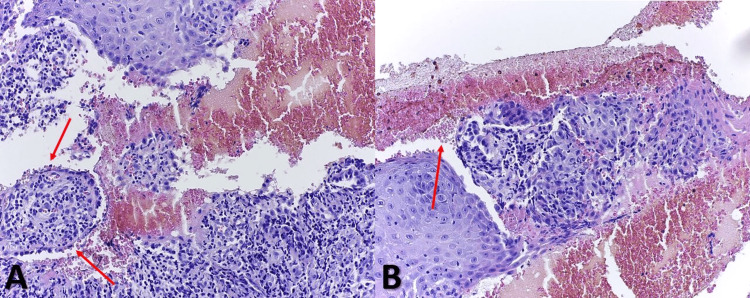
Histology examination (hematoxylin and eosin stain, 200×). Dermal papilla in the left corner of the image (red arrows) with “tombstones” basal cell keratinocytes (A) and free-floating acantholytic cells (red arrow) in the bullae content (B).

**Figure 5:**
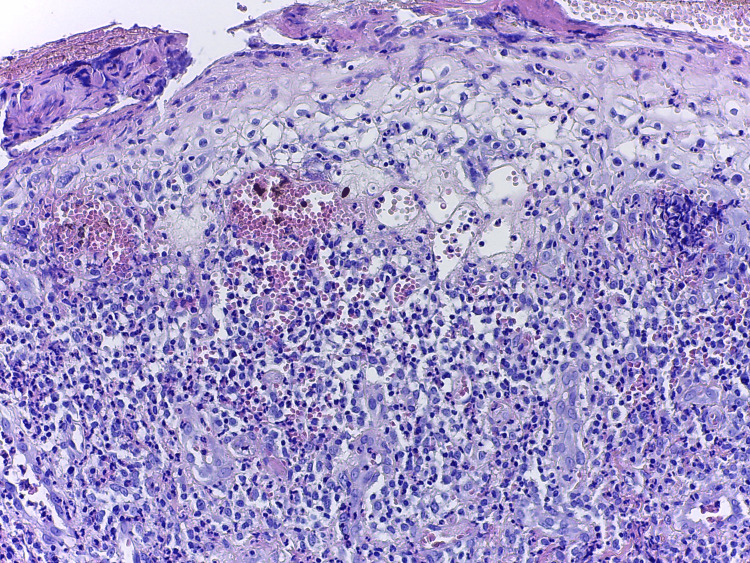
Histology examination (hematoxylin and eosin stain, 200×). Dermal spongiosis with eosinophilia, indicative of early lesion.

Laboratory examinations for autoimmune bullous diseases were performed. Indirect immunofluorescence (IIF) revealed positive anti-desmosomal antibodies at high titer (1/320; normal range: negative). Enzyme-linked immunosorbent assay (ELISA) tests for antibodies against DSG 1, DSG 3, and bullous pemphigoid antigen were all negative. Complete blood count and renal and liver tests were within the normal range. ANA was also negative when tested again in our clinic. Ophthalmology evaluation performed for the differential diagnosis with Bechet disease was normal, and no other criteria besides oral aphthous lesions were present (genital lesions, skin lesions, positive pathergy test, neurologic and vascular manifestations). As the patient refused another biopsy or DIF, a second opinion by an expert pathologist was ordered on the previous paraffin block that revealed suprabasal acantholysis (Figure 3), “tombstones” basal cell keratinocytes and free acantholytic cells in the bullae content (Figure 4), and dermal spongiosis with eosinophilia (Figure 5), concluding the diagnosis of mucous pemphigus vulgaris. There was no interface dermatitis, necrotic keratinocytes, dyskeratotic cells, or vacuolization of basal keratinocytes.

Corticosteroid therapy was started with 40 mg prednisone a day, with slow tapering and switching to methylprednisolone with good results; however, after two months of therapy, the patient reported a peripheric tremor that was bothering her considerably. Mycophenolate mofetil (MM) 1.5 g/day was added to methylprednisolone 16 mg/day, with the intent to stop corticosteroid therapy in a few months. Local dressings and antimycotic solutions were recommended. Her evolution was excellent and methylprednisolone was stopped in three months. The patient remained on MM 2 g/day for two months, followed by 1.5 g/day for another two months, with complete remission. In May 2019, she developed recurring erosive and painful oral enanthema on the tongue, gingivae, and inner inferior lip, despite being on MM treatment. Methylprednisolone 32 mg/day was quickly started and MM was increased back to 2 g/day. She remained on this treatment, with slow tapering, for six months, with complete remission in December 2019. Medrol was stopped at this point, while MM treatment continued for an additional two months. In May 2020, new erosive lesions appeared on the lateral part of the tongue, and rituximab (anti-CD23 antibodies) was considered as the next therapeutic step.

In June 2020, a routine gynecological examination with endovaginal echography revealed a hyperechogenic multiloculate tumoral mass at the inferior pole of the left ovary measuring approximately 8 cm, interpreted as a dermoid cyst. Although this was the first gynecological examination of the patient, she had undergone an abdominal ultrasound one year before which was unremarkable. An MRI showed a well-circumscribed, polylobate tumoral mass in the left iliac fossae near the left ovary measuring 9.1 × 5.8 × 8.7 cm, with T1-weighted isosignal and discrete T2-weighted hypersignal. The tumor presented a discrete cystic/necrotic component, fine septs, with a central fibrotic or calcified stellate component. Intense contrast medium retention and small lymph nodes near the iliac vessels were noted. The MRI findings suggested a dysgerminoma; mature and immature teratoma was also considered.

In October 2020, the retroperitoneal tumor was successfully excised, and the pathology report revealed stroma-rich hyaline vascular-type CD. Postoperative evolution was excellent, and the oral lesions subsided 95% under MM 2 g/day, without residual pain or dysphagia. On local examination, only discrete erythema was visible in December 2020 on the gingivae and internal inferior lip. Considering the local evolution and the fact that CD disease was treated effectively, we decided to maintain MM treatment for a few months and hold the administration of rituximab. She did not repeat the recommended follow-up MRI, but the local evolution was excellent and the patient remained free of disease and without any recurrences since stopping treatment for more than a year. The last clinical follow-up was in October 2022.

## Discussion

PNP is a relatively new disease, first reported by Anhalt et al. in 1990, defined as the association between atypical pemphigus and an underlying malignancy [6]. The term “paraneoplastic autoimmune multiorgan syndrome” is also used because patients can develop non-bullous skin lesions, pulmonary involvement, and antigen deposition in other organs [7]. Non-Hodgkin’s lymphoma (39%), chronic lymphocytic leukemia (18%), and CD (18%) are most commonly associated with PNP, although CD may be the leading cause of PNP in children and young adults, as was the case in our 19-year-old patient [8,9].

There are no universally accepted diagnostic criteria for PNP and diagnosis may be difficult to establish. Cases typically present with severe mucosal involvement with cutaneous polymorphic lesions, anti-plakin antibodies, acantholysis, keratinocyte necrosis or interface dermatitis on histology, deposition of IgG and complement in the epidermal intercellular spaces, as well as granular-linear complement deposition along the epidermal basement membrane zone on DIF testing and a concomitant malignancy [3,6].

Our patient never developed skin lesions in all four years of evolution and only presented with oral involvement. Moreover, although her stomatitis had a relapsing evolution and was severe at times, it did respond temporarily to treatment with corticosteroids and MM. PNP and pemphigus vulgaris can be very similar. Both diseases start with oral superficial erosions, but PNP stomatitis is usually more severe, with polymorphic and hemorrhagic lesions which are resistant to treatment [10]. After the onset of mucosal involvement, PNP cases usually develop polymorphic skin lesions, but there is another reported case with only oral involvement [11]. Interestingly, it is also a young patient (13-year-old) with PNP associated with CD, with a relatively long evolution (one and a half years) who was treated as oral pemphigus vulgaris before CD was diagnosed.

PNP presents with two major clinical variants, namely, bullous or lichenoid. Depending on the type of lesion biopsied, the histological findings are variable [10]. In bullous lesions, typical findings are suprabasal acantholysis and individual keratinocyte necrosis with sparse inflammatory infiltrate. Lichenoid interface dermatitis may be sometimes present even in bullous lesions. The unique association of suprabasal acantholysis with dyskeratotic keratinocytes may be highly indicative of PNP [12]. In pemphigus vulgaris, there is suprabasal acantholysis, “tombstones” basal keratinocytes, free-floating acantholytic cells in the bullae content, and a sparse inflammatory infiltrate, especially with eosinophils in early lesions [13]. In our patient’s case, the biopsy did not show interface dermatitis or keratinocyte necrosis, but only the typical histology for pemphigus vulgaris. In PNP, diagnosis may necessitate multiple biopsies [12], but our patient refused another intervention. DIF, done in another clinic before presentation, was negative, possibly due to sampling errors [14]. Studies have shown that a negative DIF does not rule out PNP [15].

In PNP, both clinical and histology findings can mimic other diseases, such as pemphigus vulgaris, erythema multiforme, Steven-Johnson syndrome, erosive lichen planus, or bullous pemphigoid [16]. This makes diagnosis challenging, especially when the concurrent malignancy is not yet diagnosed. Serological testing is very useful in the diagnosis, with anti-plakin antibodies being the most specific finding for PNP [17]. The most accurate IIF test for PNP is on rat bladder epithelium, which lacks DSG antigens and specifically evaluates anti-plakin autoimmunity, but this test was not commercially available in our facility. In our patient, IIF on monkey esophagus substrate was positive for anti-desmosome antibodies at high titer (starting titer 1/10, patient’s titer 1/340) repeatedly, but antibodies against DSG 1 and 3 (ELISA) were negative.

We initially established the diagnosis of mucous pemphigus vulgaris based on clinical presentation, histology, and IIF, which were all concurrent with this diagnosis. ELISA testing for anti-DSG antibodies was negative, but 6% of pemphigus vulgaris patients can present with such serological discrepancy and cases with autoimmunity to other desmosomal antigens exist [18,19]. Once CD was diagnosed histologically, it became obvious it was a case of PNP mimicking pemphigus vulgaris.

CD is a lymph node hyperplasia that can be unicentric or multicentric. It is predominantly a disease of the young and is mostly asymptomatic [2]. Patients present either with an enlarged lymph node or the diagnosis is an incidental finding on imaging, as in the case of our patient. The most common site for unicentric CD is the mediastinum, while retroperitoneal localization accounts for only 14% of cases. The gold standard treatment in operable disease is complete excision, which is usually curative, and life expectancy is not affected. However, CD with PNP has a worse prognosis and can be fatal when associated with bronchiolitis obliterans [20]. Our patient had a mild evolution, without pulmonary involvement. Her stomatitis, although long-lasting and recurring, was mild and at times responsive to treatment. After resection, she had an excellent evolution and achieved complete remission in a short time with systemic corticoid therapy and MM. She remained in remission one year after stopping all treatment.

## Conclusions

We present an atypical case of PNP associated with an asymptomatic CD, with a mild and long evolution, mimicking pemphigus vulgaris both clinically and histologically with mucous lesions only. The patient had a relatively good response to treatment even before tumor excision and quick complete remission after the tumor was resected. PNP diagnosis can be challenging when both clinical and histological findings mimic other bullous diseases, especially in patients with asymptomatic tumors. The mildness of oral involvement, lack of hemorrhagic lesions, response to therapy, as well as the presence of suprabasal acantholysis, “tombstones” basal keratinocytes, and free-floating acantholytic cells without interface dermatitis, necrotic, or dyskeratotic keratinocytes on histology, made PNP diagnosis challenging before the diagnosis of a complete asymptomatic CD tumor in this young patient. High awareness for PNP and CD should be kept in young patients with recurrent or long-lasting bullous lesions, and screening for asymptomatic neoplasia should be done in these cases.
